# A Simple Admission-Based Score for Early Mortality Risk Stratification in Non-Shock Sepsis: A Pilot Study

**DOI:** 10.3390/diagnostics16111623

**Published:** 2026-05-26

**Authors:** Simona Maria Borta, Romana Olivia Popetiu, Larisa Alexandra Rus, Anamaria Vîlcea, Cristina Maghera, Renata Padurean, Dragoş Vasile Nica, Adrian Silviu Crişan

**Affiliations:** 1 Department of Internal Medicine, Faculty of Medicine, “Vasile Goldiș” Western University of Arad, Bulevardul Revoluției 94, 310025 Arad, Romania; popetiur@gmail.com (R.O.P.); larisa_gal@yahoo.com (L.A.R.); anamariavilcea33@gmail.com (A.V.); 2 Arad County Emergency Clinical Hospital, Str. Andrényi Károly Nr. 24, 310037 Arad, Romania; cristinamaghera@yahoo.com (C.M.); padurean.renata@hotmail.com (R.P.); 3 Research Center in Dental Medicine Using Conventional and Alternative Technologies, Department of Prostheses Technology and Dental Materials, Faculty of Dental Medicine, “Victor Babeş” University of Medicine and Pharmacy, 9 Revolutiei 1989 Ave., 300070 Timișoara, Romania; nicadragos@gmail.com; 4 Research Center for Pharmaco-Toxicological Evaluations, Faculty of Pharmacy, “Victor Babeş” University of Medicine and Pharmacy, Eftimie Murgu Square No. 2, 300041 Timișoara, Romania; 5 The National Institute of Research—Development for Machines and Installations Designed for Agriculture and Food Industry (INMA), Bulevardul Ion Ionescu de la Brad 6, 077190 București, Romania; 6 Department of Critical Care and Emergency Medicine, “Vasile Goldiș” Western University of Arad, Bulevardul Revoluției 94, 310025 Arad, Romania

**Keywords:** sepsis, mortality, risk assessment, atrial fibrillation, creatinine, presepsin, early diagnosis

## Abstract

**Background/Objectives**: Early risk stratification in non-shock sepsis remains challenging, as patients may appear clinically stable despite ongoing deterioration. We used key variables across seven pathophysiological domains (cardiovascular, hematological, metabolic, hepatic, renal, immune, and comorbidity burden) to identify admission-based predictors of in-hospital mortality for these patients and derive a simple, clinically applicable triage score. **Methods**: This prospective pilot study included 182 adult non-shock sepsis patients transferred from the Emergency Department to the internal medicine ward in a tertiary hospital in Arad (Romania). Markers of cardiovascular (atrial fibrillation), renal (creatinine, urea), immune–inflammatory (IL-6, CRP, ESR, procalcitonin, presepsin), metabolic (FBG), hepatic (AST, ALT), and comorbidity burden (CCI) domains were analyzed using logistic and LASSO regression with bootstrap validation. **Results**: Non-survivors exhibited a significantly higher prevalence of atrial fibrillation (*p* = 0.012), as well as significantly higher creatinine, urea, IL-6, CRP, ESR, CCI, and presepsin (*p* ≤ 0.042). Although most variables, (except IL-6) were significant in univariate analysis (*p* ≤ 0.047), only atrial fibrillation (AOR = 2.31, 95% CI: 1.09–2.84, *p* = 0.021) and creatinine (AOR = 1.40, 95% CI: 1.00–1.95, *p* = 0.048) remained independent predictors in the multivariate model. LASSO regression (1000 bootstrap iterations, selection frequency ≥ 80%) confirmed their robustness. Three-parameter models combining atrial fibrillation, creatinine, and inflammatory biomarkers showed good discrimination, with the presepsin-based model achieving an AUC of 0.843, 80.0% sensitivity, 82.9% specificity, and NPV > 95% at the optimal cut-offs (creatinine > 1.49 mg/dL; presepsin > 1446 pg/mL). The Sepsis CORE score demonstrated progressive risk stratification, with mortality rising from 0% to 60.0% across score categories. **Conclusions**: The proposed score integrates cardiovascular, renal, and immune domains into a simple admission-based tool with high negative predictive value. It may support early triage and risk stratification in non-shock sepsis patients admitted to general wards, although external validation is required.

## 1. Introduction

Triggered by a dysregulated host response, sepsis is a life-threatening clinical entity involving multi-organ dysfunction [1]. This clinical condition is one of the most expensive medical emergencies [2] and affects a substantial number of individuals worldwide—an estimated 166 million cases and 21 million deaths in 2021 alone [3]. Although the burden of sepsis is likely to increase due to an aging population and the rising prevalence of multidrug-resistant pathogens, death rates are expected to decrease due to faster recognition, improved care bundle (early antibiotics/fluids), and widespread adoption of sepsis guidelines [4]. The incidence of sepsis in intensive care units (ICUs), however, remains high despite advances in its diagnosis and treatment, ranging between 25.8% and over 40% depending on presentation severity, associated comorbidities, and available resources [5]. In this context, early detection of high-risk patients and accurate stratification of mortality risk are central to the effective management of sepsis [6].

Different scoring systems are applied throughout the pathway of sepsis patients in the hospital, with the two most commonly used tools—the Sequential Organ Failure Assessment (SOFA) and its quick version (qSOFA)—derived from the Sepsis-3 definition [1]. Integrating neurological, respiratory, and cardiovascular components, the latter approach is routinely used prior to laboratory analysis to quickly identify high-risk patients with suspected infection outside the ICU [7,8]. Another score applicable for early detection and prioritization of septic patients is the National Early Warning Score 2 (NEWS2); it is based on seven variables across the same organ systems while also capturing systemic inflammatory response through body temperature [9]. On the other hand, SOFA is performed after the initial evaluation, typically following hospital admission, most often in the ICU. Considered the gold standard for assessing organ failure severity and predicting death risk in these settings, this score measures the degree of dysfunction across six key organ systems (respiratory, cardiovascular, hepatic, coagulation, renal, and neurological) on a 0–4 scale, with a change from baseline indicating sepsis in patients with confirmed infection [7,8]. Alternative assessment tools used in the ICU context are the Acute Physiology and Chronic Health Evaluation II (APACHE II) score and the Simplified Acute Physiology Score II (SAPS II). These models are applied within 24 h of ICU admission, but are highly complex, incorporating more than 15 variables [10]. Following admission to general wards from the Emergency Department (ED), early warning scores such as National Early Warning Score 2 (NEWS2) or Modified Early Warning Score (MEWS) are used for continuous monitoring of clinical deterioration, while SOFA may be applied when laboratory data are available [11]. However, these tools are not specifically designed for mortality risk prediction at admission to general wards.

A highly dynamic condition, sepsis can evolve rapidly; thus, patients admitted to the ED and then allocated to the general ward may deteriorate within hours [1,12,13]. Non-ICU sepsis patients represent less severe presentations, but constitute the majority of cases, with most of them (up to two-thirds) managed outside the ICU [14]. Their in-hospital death rates, unfortunately, may approach one-third, which is comparable to ICU rates [14]. Accurate risk stratification at general ward admission therefore represents a critical opportunity to optimize triage decisions, resource allocation, and timely escalation of care in non-shock sepsis patients. In contrast, dedicated admission-based prognostic tools for this specific population remain limited. We therefore hypothesized that admission-available variables reflecting dysfunction across seven major pathophysiological domains (cardiovascular, hematological, metabolic, hepatic, renal, immune, and comorbidity burden) could facilitate early identification of patients at increased risk of in-hospital mortality. Importantly, such patients may appear clinically stable at admission despite already harboring biological signs of impending deterioration. Atrial fibrillation (AF) was included as a marker of cardiovascular vulnerability and hemodynamic stress since cardiac rhythm complications (especially AF) are not well integrated into sepsis prognostic scores, despite their established links with mortality and post-discharge events [15,16,17,18,19]. Creatinine and urea are embedded in organ dysfunction phenotyping and correlate with systemic hypoperfusion and multi-organ reserve loss, and, therefore, were applied as proxies for sepsis severity [20,21,22,23,24,25]. A comprehensive inflammatory panel included interleukin-6 (IL-6), C-reactive protein (CRP), and erythrocyte sedimentation rate (ESR) as complementary metrics capturing upstream cytokine signaling, acute-phase activation, and broader systemic inflammation; leukocyte count as an indicator of cellular immune response; procalcitonin as a biomarker reflecting the host response to bacterial infection; and presepsin as an early indicator of innate immune activation [26,27,28,29,30,31]. Fasting blood glucose was used as a surrogate marker of stress-induced metabolic dysregulation, while aspartate aminotransferase (AST) and alanine aminotransferase (ALT) served as indirect measures of hepatocellular injury and potential hypoxic or inflammatory liver involvement in sepsis [32,33]. A structured summary of chronic disease burden, the Charlson Comorbidity Index was utilized as a measure of baseline vulnerability [34]. The results are relevant for clinicians and healthcare systems aiming to optimize patient flow and resource allocation outside the ICU. They also provide a basis for developing and validating simplified, clinically useful risk scores tailored to non-ICU sepsis populations.

## 2. Materials and Methods

### 2.1. Study Design and Setting

This single-center pilot study involved a prospective cohort design to determine baseline variables that best predict in-hospital mortality in patients with sepsis admitted from the ED to the internal medicine ward. The study was conducted at the Emergency Clinical County Hospital (ECCH) Arad, Romania. With over 1300 beds, this tertiary care hospital serves a population of approximately 400,000–450,000 inhabitants from Arad County and surrounding regions and includes specialized departments, such as internal medicine and ICUs [35].

This exploratory study was conducted in accordance with the Declaration of Helsinki 1964 and later amendments. The protocol was approved by the Ethics Committee (IEC) of ECCH (approval No. 77/approval date: 30 April 2024). The study population (182 patients) was selected from sepsis patients admitted to this hospital between 10 May 2024 and 28 November 2025. Sepsis was defined as per the Sepsis-3 criteria—suspected or confirmed infection associated with an acute increase of ≥2 points in the SOFA score [1]. We recruited only individuals in early sepsis (not yet in septic shock) as their risk profiles may be more concerning in general wards because their deterioration is less obvious and more easily missed [36]. A binary outcome (dead/alive) was used for in-hospital mortality to align with the study objective of developing a rapid triage tool based on admission variables, where immediate risk stratification is clinically more relevant than time-to-event estimation. All patients or their caregivers received and signed informed consent.

### 2.2. Inclusion and Exclusion Criteria

**Figure 1:**
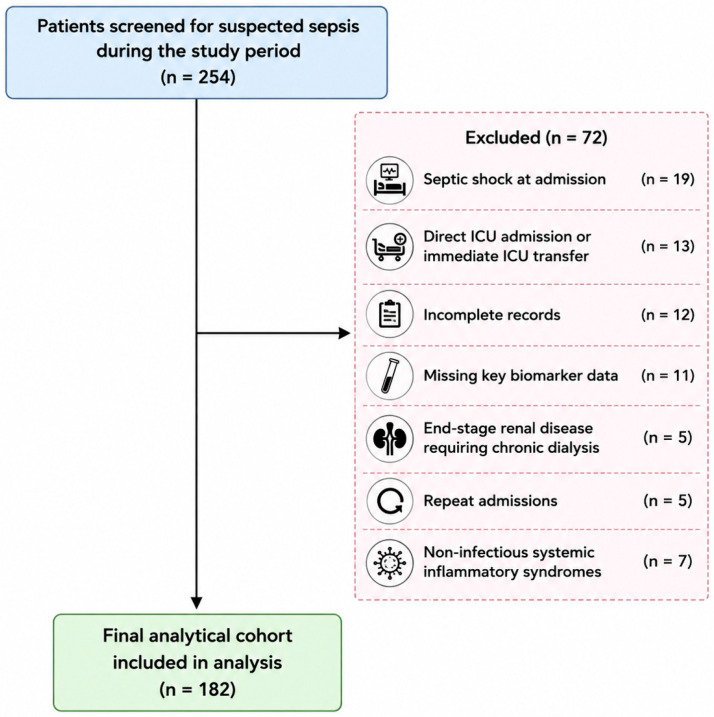
Patient selection flowchart for cohort derivation. A total of 254 patients with suspected sepsis were initially screened during the study period. Seventy-two patients were excluded due to septic shock at admission, direct ICU admission or immediate ICU transfer, incomplete medical records, missing key biomarker data, end-stage renal disease requiring chronic dialysis, repeat admissions, or non-infectious systemic inflammatory syndromes. Following application of all inclusion and exclusion criteria, the final analytical cohort consisted of 182 non-shock sepsis patients transferred from the Emergency Department to the internal medicine ward for analysis.

The primary inclusion criteria were as follows: (i) adults aged 18 years and older diagnosed with sepsis as defined according to the Sepsis-3 criteria; (ii) sepsis patients transferred to the internal medicine ward after ED admission with documented sepsis management protocols; (iii) first sepsis admission only; (iv) admission variables available within the first 24 h; (v) availability of comprehensive sociodemographic and clinical data; and (vi) provision of informed consent by the patient or legal representative. We restricted the analysis to patients admitted to internal medicine wards to ensure a homogeneous non-ICU population and to specifically address early risk stratification in settings with lower monitoring intensity. The patient selection flowchart for cohort derivation is illustrated in Figure 1.

Exclusion criteria were as follows: (i) incomplete medical records; (ii) patients with septic shock; (iii) patients with other critical illnesses or sepsis of non-infectious origin, such as acute pancreatitis, major trauma, postoperative systemic inflammation, autoimmune flare, or drug-induced systemic inflammatory response; (iv) patients with a do-not-resuscitate (DNR) order in place at the time of admission; (v) patients directly admitted to the ICU or requiring immediate ICU transfer; (vi) repeated hospital admissions of the same patient (only the first episode was included); and (vii) patients with end-stage renal disease requiring chronic dialysis.

### 2.3. Sociodemographic and Laboratory Variables

Chronic kidney disease was defined as the presence of kidney damage (e.g., albuminuria, structural abnormalities) and/or a sustained reduction in kidney function expressed as estimated glomerular filtration rate (eGFR) of less than 60 mL/min/1.73 m^2^ lasting 3 months or more [37]. Diabetes mellitus was considered present if documented in the medical records based on a prior clinical diagnosis, fasting plasma glucose ≥ 126 mg/dL (7.0 mmol/L), HbA1c ≥ 6.5%, or current use of antidiabetic medication (oral agents or insulin) [38]. Septic ED patients with atrial fibrillation were grouped together irrespective of temporal onset (pre-existing versus new) because they may share overlapping pathophysiological mechanisms, have similar immediate clinical consequences, and are hard to classify accurately at presentation [15,16,17]. The majority of AF cases were clinically consistent with pre-existing arrhythmia, although precise subtype separation was limited by pilot cohort documentation constraints. This approach was used to enhance statistical robustness, improve model stability, and reduce the risk of spurious associations in the context of the limited sample characteristic to pilot studies.

Biochemical analyses were performed during the initial 24 h of hospital admission. Total white blood cell count was determined from EDTA-anticoagulated blood with the SYSMEX XN-1500 analyzer (Sysmex Corporation, Kobe, Japan). Liver transaminases (aspartate aminotransferase and alanine aminotransferase) were determined by standardized enzymatic assays according to the recommendations of the International Federation of Clinical Chemistry and Laboratory Medicine (IFCC), using an automated clinical chemistry analyzer (COBAS c 501, Roche Diagnostics, Mannheim, Germany). Fasting glucose as well as serum urea and creatinine were measured on the same platform.

Serum IL-6 and procalcitonin levels were analyzed using electrochemiluminescence immunoassays on the COBAS e 601 platform (Roche Diagnostics, Mannheim, Germany). CRP was determined via the COBAS b 101 system (Roche Diagnostics, Mannheim, Germany), whereas erythrocyte sedimentation rate was measured using the Westergren method. Presepsin concentrations were measured using a chemiluminescent enzyme immunoassay on a PATHFAST analyzer (LSI Medience Corporation, Tokyo, Japan). Patient samples and calibration standards were assayed in triplicate to ensure analytical reproducibility and precision. Internal quality controls were included in each analytical run, and external quality assessment was performed in accordance with laboratory accreditation protocols.

### 2.4. Statistical Analysis

We first assessed intergroup differences in age using a Mann–Whitney U test with Benjamini–Hochberg correction for multiple testing. Chi-square (χ^2^) tests based on 2 × 2 contingency tables—or Fisher’s exact tests (for small sample size)—were applied to compare the distribution of sex, origin area, chronic kidney disease, atrial fibrillation, and diabetes between survivors and non-survivors. We next used Mann–Whitney U tests with Benjamini–Hochberg correction to determine whether significant differences exist between groups for leukocytes, fasting blood glucose, aspartate aminotransferase, alanine aminotransferase, urea, creatinine, interleukin-6, CRP, erythrocyte sedimentation rate, procalcitonin, Charlson Comorbidity Index, and presepsin at admission. Significant variables were considered biologically and clinically responsive to disease progression and treatment, and therefore more likely to reflect sepsis severity and systemic impact.

Univariate logistic regression was applied to significant comorbidity-related categorical variables and continuous variables. Independent predictors were subsequently included in the multivariate logistic regression model. All-cause in-hospital mortality was used, including deaths occurring after escalation to ICU. To improve transparency regarding model robustness, we also quantified event burden and modeling constraints. Thirty mortality events were available for multivariable modeling, corresponding to an events-per-variable (EPV) ratio of approximately 4.3 in the initial candidate model prior to penalization. Patients with incomplete predictor data were excluded prior to modeling, and complete-case analysis was performed. Given the exploratory pilot design and inherent overfitting risk associated with this modest ratio, the Least Absolute Shrinkage and Selection Operator (LASSO) regression was applied in parallel with conventional logistic regression for variable selection, reduction in model overfitting, and mitigation of multicollinearity among predictors by shrinking less informative coefficients toward zero [39]. Stability of variable selection in the LASSO model was assessed using bootstrap resampling (1000 iterations), with predictors selected in at least 80% of resamples being considered robust. To enhance model robustness, only variables confirmed as independent predictors by both logistic regression and LASSO were included in the final analysis. If the combination of these variables did not meet a predefined performance threshold, i.e., ≥0.8 for both sensitivity and specificity, combinations with additional variables were evaluated to improve diagnostic performance. Continuous predictors were z-standardized for exploratory univariate logistic regression analyses to allow direct comparison of effect sizes across heterogeneous biomarkers. For final multivariate logistic regression, predictors were entered using their original clinical units to preserve clinical interpretability.

Combinations of variables were limited to a maximum of three to avoid model overfitting and preserve interpretability. The aforementioned cut-off was selected a priori to indicate good discriminative performance using commonly accepted benchmarks for good diagnostic accuracy reported in the literature [40]. The performance of these three-parameter models was further evaluated using the DeLong test to compare the areas under curve (AUC) between models and Nagelkerke pseudo-R^2^ to estimate explained variability. Calibration was assessed using the Hosmer–Lemeshow test, although formal calibration slope and optimism correction analyses were not available. These metrics provide complementary information on discrimination, model strength, and goodness-of-fit. A score was derived from the combination of variables that provided the best balance between sensitivity and specificity. The cut-off thresholds for continuous variables were established through Receiver operating characteristic (ROC) curve analysis, using the Youden index to maximize accuracy. A *p*-value less than 0.05 was considered significant [41]. Statistical analyses were performed using IBM SPSS Statistics for Windows, Version 23.0 (IBM Corp., Armonk, NY, USA), with additional penalized regression analyses conducted using R version 4.4.1 (R Foundation for Statistical Computing, Vienna, Austria). 

## 3. Results

The median age of the study population was 71 years (66; 78). This cohort exhibited a balanced sex ratio—with 90 males (49.50%) and 92 women (50.50%), but a predominantly urban origin—with 104 patients (57.14%) from urban areas and 78 patients (42.86%) from rural areas. Bacterial infections were most prevalent in patients with sepsis, being detected in 102 individuals (56.7%). Fungal infections (mycoses), by contrast, were infrequently encountered, accounting for only ten cases (5.5%). Approximately 37.8% of patients (68 out of 182 cases) represented suspected or culture-negative sepsis cases, reflecting the absence of microbiological confirmation despite compatible clinical presentation. Chronic kidney disease was the most common comorbidity, occurring in 80 patients (44%); atrial fibrillation was present in 54 patients (29.70%) and diabetes in 66 patients (33.60%).

### 3.1. Baseline Biochemical Profile by Survival Status

Demographic profiles and comorbidity distribution in patients stratified by survival status (survivors versus non-survivors) are provided in Table 1. There were no significant intergroup differences in mortality according to sex (χ^2^ test, *p* = 0.604), origin area (χ^2^ test, *p* = 0.997), chronic kidney disease status (χ^2^ test, *p* = 0.278), or diabetes status (χ^2^ test, *p* = 0.226). However, a significantly higher proportion of non-survivors was observed among individuals with atrial fibrillation (χ^2^ test, *p* = 0.012).

Median values for demographic data (age), clinical comorbidity burden (Charlson Comorbidity Index), and laboratory parameters (fasting blood glucose, aspartate aminotransferase, alanine aminotransferase, interleukin-6, C-reactive protein, erythrocyte sedimentation, procalcitonin, and presepsin) in patients stratified by survival status are shown in Table 2. Patients who improved their clinical condition were of similar age to those who died. Most variables exhibited markedly abnormal laboratory values at admission irrespective of survival status, with the exception of hepatic parameters. Surviving patients, however, showed significantly lower urea, creatinine, IL-6, CRP, erythrocyte sedimentation rate, Charlson Comorbidity Index, and presepsin. No significant differences were observed for the other parameters analyzed.

### 3.2. Univariate, Multivariate, and LASSO Regression Analysis for Mortality Prediction

The results of the univariate, multivariate, and LASSO regression analyses are presented in Table 3. Based on the results from Table 1 and Table 2, urea, creatinine, IL-6, CRP, erythrocyte sedimentation rate, Charlson Comorbidity Index, presepsin, and the presence of atrial fibrillation were selected for inclusion in the univariate logistic regression model. It was found that, except for interleukin-6, all the parameters serve as (unadjusted) predictors of in-hospital mortality. After the application of multivariate logistic regression on these parameters, atrial fibrillation and creatinine remained the only statistically significant predictors. The presence of atrial fibrillation was associated with an almost twofold increase in the odds of the outcome. Creatinine also showed a borderline association with the risk of death in the multivariate model, whereas the other parameters showed minimal, non-significant effects.

Table 4 shows the comparative analysis of regression models developed for predicting survival in sepsis. The presence of atrial fibrillation alone revealed a modest discriminative capacity, whereas addition of serum creatinine on admission yielded a substantial increase in model performance. The highest discriminative ability was found for three-parameter models incorporating these parameters and either CRP or presepsin. The mathematical reliability of the proposed models was supported by the results of the Hosmer–Lemeshow test, where all variants revealed an acceptable calibration between the predicted probabilities and the observed deaths. The model including presepsin exhibited the best calibration and the highest global accuracy, with nearly half of the mortality variation being explained by the interaction of the three selected factors.

### 3.3. The Sepsis CORE Score

All three-parameter models given in Table 4 showed an excellent negative predictive value, underlining the usefulness of the score as a triage tool—the absence of risk factors (score 0) allowed the exclusion of death with a negative predictive value exceeding 95%. While the model including erythrocyte sedimentation rate maximized the sensitivity, the inclusion of presepsin optimized the specificity, markedly reducing the incidence of false alarms and facilitating a more accurate identification of patients requiring intensive care. We next transformed continuous variables into ordinal score points using ROC-derived cut-off values—given in Table 5. The distribution of mortality risk according to score points is given in the same table.

## 4. Discussion

Although clinicians rely on robust, validated metrics for managing sepsis [42], the heterogeneous nature and rapid progression of this life-threatening condition often render these indices insufficient for timely clinical decision-making and capturing individualized pathophysiological profiles [43,44]. This is among the few studies specifically targeting sepsis patients transferred to general wards after ED admission—a distinct, heterogeneous, intermediate-risk subgroup. Our cohort included predominantly older adults, with similar profiles in terms of sex, origin area, chronic kidney disease, and diabetes across survival groups, and, therefore, mortality within our study population may reflect acute pathophysiological processes rather than pre-existing conditions. A substantial proportion of cases represented culture-negative or clinically suspected sepsis, reflecting real-world diagnostic complexity. This diagnostic heterogeneity may have influenced biomarker performance, particularly for infection-responsive inflammatory markers, as microbiological confirmation was unavailable in a substantial subset of patients. Therefore, variability in underlying infectious burden or pathogen type should be considered when interpreting biomarker associations and may affect broader generalizability.

The prevalence of atrial fibrillation was significantly higher in non-surviving septic patients transferred from the ED to the internal medicine ward, and this variable was the most robust independent predictor in both multivariate logistic regression and LASSO models. This is directionally consistent with a substantial literature supporting its role as a clinically meaningful determinant of increased mortality and adverse clinical outcomes, irrespective of whether pre-existing or newly developed [15,16,18,19]. Nevertheless, some large database studies employing more rigorous balancing strategies have demonstrated attenuation of this association [17]. It is important to note that these studies (and most of the recent literature) have focused on new-onset atrial fibrillation, and as a consequence, may underestimate the overall burden and prognostic impact of atrial fibrillation in septic patients by excluding those with pre-existing arrhythmia. Taken together, these data support the plausibility of atrial fibrillation as a mortality signal for sepsis patients admitted to internal medicine wards following ED admission. These outcomes may be related to the hemodynamic instability, heightened physiological stress, and autonomic dysfunction associated with this cardiac condition [19].

At the same time, atrial fibrillation should not necessarily be interpreted as a direct causal determinant of mortality but rather may reflect a broader composite marker of cardiovascular vulnerability, frailty burden, and acute systemic stress. In the present pilot cohort, pre-existing and new-onset atrial fibrillation were analyzed collectively due to sample size limitations and triage-focused design. Therefore, mechanistic distinctions between chronic arrhythmic burden and acute sepsis-induced electrophysiological dysfunction could not be fully evaluated. Separating pre-existing from new-onset atrial fibrillation may be important in future studies because these subtypes could reflect distinct underlying mechanisms and clinical implications. Pre-existing AF may primarily indicate chronic cardiovascular disease, frailty burden, and baseline comorbidity, whereas new-onset AF may more directly reflect acute sepsis-induced electrophysiological instability and systemic inflammatory severity. Distinguishing these groups could therefore improve mechanistic understanding, refine prognostic precision, and determine whether subtype-specific weighting enhances predictive model performance.

Comparable white blood cell counts across survival groups are consistent with common clinical experience: leukocytosis (elevated leukocyte count) is frequent in bacterial sepsis but is neither sensitive nor specific for infection severity, whereas trajectories (e.g., persistent leukocytosis, delayed normalization, or leukopenia) may provide greater prognostic value than a single baseline measurement [29]. We note that fasting glucose and hepatic transaminases were similar between survivors and non-survivors. Indeed, stress hyperglycemia metrics and non-linear relationships may outperform raw glucose for mortality prediction, especially when diabetes and kidney injury interact. This may explain why baseline glucose alone often fails to distinguish survival in mixed sepsis cohorts [33]. On the other hand, clinically meaningful hepatic dysfunction in sepsis usually appears as cholestasis or hypoxic hepatitis during shock, rather than as uniformly elevated De Ritis ratios. Without bilirubin, International Normalized Ratio (INR), and shock physiology, this ratio alone often undercaptures the hepatic phenotype [32].

Although urea was significantly associated with in-hospital mortality in the univariate analysis, its effect disappeared after adjustment, likely reflecting its close correlation with renal function markers such as creatinine [24], which may better capture the underlying pathophysiological burden [45]. Significantly higher urea and creatinine observed in non-surviving patients, however, hint at renal functional decline as one of the earliest and most robust biochemical signals of a fatal outcome in this cohort. This is in line with evidence derived from studies on critically ill sepsis patients admitted to the ICU [21,23,24,25]. It may be tempting to interpret these findings as reflecting isolated reduction in glomerular filtration capacity, but existing evidence suggests a combination of renal dysfunction and systemic metabolic alterations as the most plausible cause. Specifically, the sepsis-related inflammatory response may cause the rapid and severe breakdown of skeletal muscle, leading to an increase in nitrogen load processed by the liver into urea and resulting in the subsequent rise in serum urea concentrations [46,47]. The increased reabsorption of water and sodium in states of reduced effective circulating volume or “subclinical” hypoperfusion may also account for this outcome [48].

Similarly, the existing literature reveals significant positive associations between elevated serum creatinine, disease severity, and reduced survival in sepsis patients, with data predominantly drawn from ICU populations [20,22,49]. The strong association between creatinine and in-hospital mortality in the univariate analysis retained statistical significance in the multivariate model. It is important to note that the inclusion of creatinine in the LASSO regression model supports its continued relevance as a predictor—penalized approaches tend to retain variables with stable predictive contribution even in the presence of multicollinearity [39]. This finding suggests that, although its independent effect is partially absorbed by correlated covariates in conventional regression, creatinine remains an integral component of the overall prognostic profile. Therefore, its marginal significance in multivariate analysis should not be interpreted as a lack of clinical importance, but rather as evidence of shared explanatory pathways with other severity-related variables. Nonetheless, abnormal serum creatinine may reflect underlying chronic kidney disease [50]. Several studies suggest a complex relationship between creatinine and mortality, with several factors—including the timing and severity of elevation—functioning as moderators of these associations. A 0.3 mg/dL increase in serum creatinine is generally considered a clinically relevant threshold for early identification of high-risk sepsis patients [51], especially for persistently elevated values within the first three days following ICU admission [52,53]. This is not universally applicable across all patient populations as this relationship may be modified by factors such as muscle mass, nutritional status, and the underlying cause of acute kidney injury [54,55]. As a result, it is important to consider creatinine in the broader clinical context of sepsis rather than in isolation.

The prognostic impact of inflammatory biomarkers, as observed in the univariate analysis, was not maintained after adjustment, probably due to their shared variance. Preceding hepatic CRP production, IL-6 is an early pleiotropic cytokine released following innate immune activation in early sepsis, peaking early and declining quickly [31]. We collected samples within the first 24 h post-admission, likely capturing, at least in part, this early inflammatory surge. Significantly lower baseline IL-6 levels observed here in sepsis survivors are congruent with previously reported data [56,57]. Interestingly, age can modify the association of IL-6 with mortality, with strong correlations seen in non-elderly ICU patients but not in elderly patients—where baseline IL-6 was high even among survivors [58].

An hepatic IL-6–driven acute-phase protein, CRP rises within 6–12 h after inflammatory onset and peaks at 24–48 h; this limits its utility for hyper-early discrimination at presentation [27]. This inference is supported by the strength of statistical evidence for the difference between groups, relative to IL-6. A recent review found that the prognostic value of CRP in sepsis remains uncertain due to limited low-quality evidence [59]. In fact, dynamic CRP features (e.g., CRP velocity or change over 48–72 h) typically outperform baseline CRP in ICU studies, supporting the idea that trajectory matters more than a single value [60].

In contrast to the aforementioned inflammatory metrics, erythrocyte sedimentation rate rises slowly (often days) and falls slowly [30]. This pattern may account for the marginal statistical significance of the difference observed between survivors and non-survivors. Since it is affected by multiple factors (e.g., anemia, age, sex, malignancy, autoimmune disease, chronic kidney disease), erythrocyte sedimentation rate can be elevated even in the absence of acute infection [30]. This renders it particularly vulnerable to “comorbidity-reserve confounding” in ward populations.

Similar procalcitonin levels observed in our cohort between survival groups may be attributed to procalcitonin being primarily associated with bacterial infections [28]. As a result, this marker may fail to adequately capture inflammation in less severe sepsis. Indeed, procalcitonin levels measured within the first 24 h serve as a poor 28-day mortality predictor in severe sepsis/septic shock populations [61].

The Charlson Comorbidity Index was initially associated with mortality; despite this, its effect was attenuated after adjustment, suggesting mediation by acute clinical/biological parameters included in the model. This index plausibly reflects physiologic reserve, frailty, and treatment limitations in older sepsis patients, with several studies supporting independent prognostic value for short-term survival [62,63]. Furthermore, chronic disease burden and malignancy are key determinants of non-ICU sepsis mortality [64]. The lower Charlson Comorbidity Index in survivors hence likely captures two realities: less baseline organ dysfunction and lower probability of treatment limitation.

Presepsin reflects monocyte/macrophage activation and can rise early in bacterial sepsis. It is also strongly affected by renal function and age; baseline presepsin increases as estimated glomerular filtration rate declines [26]. Elevated presepsin observed here in non-survivors may reflect increased immune activation, higher bacterial burden, and reduced renal clearance. Given the similar age distribution between groups, the influence of age on renal function is likely limited. Our findings are consistent with prior studies reporting greater presepsin concentrations in non-survivors, supporting its role as a potentially relevant mortality-associated biomarker in sepsis [65,66].

Despite modest association with mortality in the univariate analysis and lack of independent association in the multivariate model, the inclusion of presepsin in a model including atrial fibrillation and serum creatinine resulted in the best calibration and highest overall predictive accuracy, with a substantial proportion of mortality variation explained by the interaction of these variables. As a soluble CD14 subtype released during monocyte activation in response to bacterial components, presepsin is closely linked to innate immune signaling pathways that overlap with those captured by other inflammatory biomarkers [66], thereby limiting its independent contribution in conventional regression models. This finding provides evidence that presepsin provides complementary rather than redundant prognostic information, especially integrated into multivariable frameworks that reflect complementary prognostic domains of the septic response. However, practical implementation of this biomarker may be constrained by assay cost, laboratory infrastructure requirements, and variable response times across institutions, although this marker enhanced model specificity and overall discriminative performance. These limitations may be particularly relevant in resource-limited settings or during urgent Emergency Department triage. In such scenarios, the simplified AF + creatinine model may represent a more pragmatic alternative, offering readily available, low-cost admission variables with good predictive performance, albeit with somewhat reduced specificity. As a result, presepsin-enhanced Sepsis CORE may be best viewed as an optimized model for higher-resource environments, whereas simplified variants may improve broader feasibility.

From an initial multidomain framework, the final model converged toward three key prognostic axes: cardiovascular, renal, and immune. This three-item architecture is familiar to clinicians and resembles the ergonomics of the qSOFA, which reduces the risk of errors and facilitates clinical implementation in triage [1]. The creatinine cut-off (>1.49 mg/dL) fits well into the context of existing scores. For example, the range 1.2–1.9 mg/dL corresponds in SOFA to one point per renal component [67]. The STM threshold is within this range and, in clinical significance, targets mild–moderate renal dysfunction that already counts as organ dysfunction. Moreover, this value lies close to the clinically relevant threshold (1.5 mg/dL) that appears recurrently in KDIGO criteria for acute kidney injury (as a ratio to baseline) [68]. On the other hand, the threshold proposed for presepsin is close to benchmark values reported in major clinical trials. In a prospective multicenter ED study, Ulla et al. showed that presepsin was approximately 875 pg/mL in sepsis, increasing to 1460 pg/mL in severe sepsis/septic shock [69]. In addition, Baik et al. reported a cut-off of ~1898.5 pg/mL for sepsis-induced mortality in a small validation study (40 patients), with broadly similar specificity and sensitivity [66].

Overall, the Sepsis CORE Score represents a candidate risk stratification model in non-shock sepsis patients since it allows a rapid rule-out of patients with low-risk profiles (those scoring 0), reduction in false alarms by including presepsin, and integrates a cardiovascular component associated with worse outcomes in sepsis. In principle, this score may help partially bridge the gap between SOFA and qSOFA by remaining concise in structure while potentially offering greater biological context than qSOFA, with thresholds informed by presepsin literature and the renal SOFA/KDIGO framework [38]. Notably, the multivariate odds ratios for atrial fibrillation and serum creatinine appear sufficiently stable for exploratory purposes, particularly given their consistent retention across conventional multivariate logistic regression, LASSO penalization, and bootstrap validation procedures. This concordance across complementary modeling strategies supports the possibility that these predictors may represent clinically meaningful mortality signals, although confirmation in larger external cohorts is required.

The results of our study contribute to the current understanding of sepsis assessment in three main areas. First, atrial fibrillation is treated as an important prognostic feature rather than as an incidental complication, and in this cohort showed the strongest association within the multivariable prediction. Prior sepsis literature often studies AF in isolation (incidence/outcomes) rather than embedding it into minimal, bedside-usable mortality scores. Second, this three-item score yielded a strong rule-out signal, with a score of 0 corresponding to no observed mortality, and all three-parameter models showed a high reliability for identifying low-risk patients. In practice, triage tools with high negative predictive value (NPV) can be clinically valuable for identifying patients unlikely to die in-hospital in that setting, helping prioritize monitoring intensity or ICU consideration for higher-score strata. However, this must be validated externally because NPV depends heavily on baseline mortality prevalence. Third, presepsin was used as a specificity-optimizing third feature, with the SMST achieving the highest specificity and the best overall accuracy among the three-parameter variants. In this context, our work proposes a clinically relevant cut-off (1446 pg/mL) in an elderly, high-CKD sepsis cohort—a subgroup where presepsin cut-offs are particularly uncertain.

While our findings are encouraging, several limitations should be noted. One important limitation of this study is the relatively modest sample size and limited number of mortality events, which resulted in a borderline events-per-variable ratio and may have reduced multivariable statistical power while increasing susceptibility to coefficient instability, model optimism, and potential overfitting. Although this constraint is inherent to pilot cohorts, we implemented several methodological safeguards to improve robustness, including LASSO penalization, bootstrap resampling, and bootstrap selection frequency thresholds to identify stable predictors and reduce coefficient inflation. Furthermore, the final Sepsis CORE score was intentionally restricted to a parsimonious three-variable structure in order to preserve interpretability while minimizing model complexity. Importantly, our primary objective was triage-oriented early risk stratification rather than definitive causal inference, and simplified models may still provide clinically meaningful preliminary utility when appropriately interpreted. Nevertheless, given the statistical limitations associated with the modest events-per-variable ratio, the Sepsis CORE score should be regarded as an early-stage derivation model requiring larger multicenter external validation cohorts before broader clinical implementation.

It is important to acknowledge that we conducted this study as a single-center pilot derivation cohort within a tertiary internal medicine setting. This strategy may not fully reflect broader sepsis populations across diverse healthcare systems or critical care pathways. Patients admitted to general medical wards after Emergency Department evaluation may also differ substantially from ICU cohorts, septic shock populations, or healthcare systems with alternative triage structures, thereby potentially affecting both baseline mortality risk and prognostic variable performance. Regional differences in microbiological epidemiology, antimicrobial resistance patterns, healthcare infrastructure, laboratory turnaround times, and biomarker assay availability may further impact the transportability of the proposed score across institutions. In particular, the practical accessibility and cost-effectiveness of presepsin testing may vary considerably between high-resource and resource-limited settings, potentially influencing real-world implementation feasibility.

Another important limitation is the potential renal confounding of presepsin, particularly in the context of a high CKD prevalence in the study population. Our combined models include creatinine, partially offsetting renal confounding. One could also argue that the prognostic value of presepsin in this context lies in integrating immune activation and renal vulnerability—both clinically relevant in sepsis [24]. The apparent absence of mortality should be interpreted with caution since the observed “perfect rule-out” performance may not be reproducible, as zero-event cells are common in small cohorts. However, this finding may be more appropriately framed as indicating a very low observed risk in this cohort, rather than implying complete absence of risk. On the other hand, the relatively advanced age of this cohort and the high prevalence of chronic kidney disease may have significantly influenced both mortality dynamics and biomarker thresholds, particularly for renal-sensitive variables such as creatinine and presepsin. Because chronic renal dysfunction may independently elevate presepsin concentrations, direct extrapolation of the present score thresholds to younger populations or cohorts with lower CKD prevalence should be approached cautiously. Future validation studies should therefore assess subgroup-specific recalibration across diverse renal function and age strata.

Moreover, direct head-to-head comparison with established prognostic tools such as qSOFA, NEWS2, MEWS, and SOFA was not feasible within the present pilot cohort due to incomplete availability of all standardized physiological variables required for accurate retrospective score reconstruction. The incremental prognostic value of Sepsis CORE relative to these established systems therefore remains uncertain and should be prioritized in future multicenter validation studies. Finally, the exclusion of septic shock patients may limit the generalizability of the findings to critically ill populations, but it allowed for a more homogeneous cohort and reduced confounding by disease severity.

## 5. Conclusions

We propose the Sepsis CORE score as a pragmatic tool for early mortality risk stratification in non-shock sepsis patients transferred from the ED to general wards after initial evaluation. This pathophysiology-based triage tool integrates atrial fibrillation, serum creatinine, and presepsin, thus capturing three critical domains of sepsis progression: cardiovascular instability, organ dysfunction, and dysregulated host immune response. Although several metrics were associated with mortality in univariate models, only atrial fibrillation and serum creatinine remained independent predictors after multivariate adjustment and application of penalized regression. These parameters revealed strong positive associations with in-hospital mortality, supporting their potential role as markers of systemic severity. The addition of presepsin yielded a clinically relevant improvement in model performance, leading to the best calibration and highest overall predictive accuracy among the three-parameter models. These findings indicate that immune activation provides complementary prognostic information beyond clinical variables alone.

With a particularly high negative predictive value, the Sepsis CORE score may reliably exclude short-term mortality in the absence of the identified risk factors. This renders it a valuable triage tool in emergency and critical care settings. Furthermore, the progressive increase in mortality across score categories supports its utility for dynamic risk stratification and decision-making, ranging from standard monitoring to early intensive care escalation. Compared with established prognostic systems such as SOFA, qSOFA, NEWS2, or MEWS, which incorporate broader physiological monitoring and dynamic organ dysfunction assessment, the Sepsis CORE score was designed as a simplified admission-based exploratory framework focused specifically on early mortality risk stratification in non-shock sepsis patients transferred to general wards. Derived from a limited set of clinically accessible cardiovascular, renal, and immune variables, this model may offer practical advantages in rapid triage contexts, particularly during initial patient evaluation or in settings where comprehensive scoring may be less feasible. However, it should not be interpreted as a replacement for established sepsis assessment tools.

## Figures and Tables

**Table 1:** Demographic characteristics and comorbidities of survivors and non-survivors.

**Sex**			**Origin Area**			
	Survivors	Non-survivors		Survivors	Non-survivors	
Male	78 (86.67%)	12 (13.34%)	Urban	86 (82.69%)	18 (17.31%)	
Female	76 (80.43%)	18 (19.56%)	Rural	66 (84.62%)	12 (15.38%)	
	**Chronic Kidney Disease**		**AF**		**Diabetes**	
	Survivors	Non-survivors	Survivors	Non-survivors	Survivors	Non-survivors
No	90 (88.23%)	12 (11.78%)	116 (90.62%)	12 (9.38%)	102 (87.93%)	14 (12.07%)
Yes	62 (77.60%)	18 (22.40%)	36 (66.67%)	18 (33.34%)	50 (75.75%)	16 (25.25%)

AF, atrial fibrillation. Because precise differentiation between pre-existing and new-onset AF was limited, AF was analyzed as a combined variable to preserve statistical power. Data are shown as absolute values with the corresponding percentages in parentheses.

**Table 2:** Selected baseline parameters at admission in survivors and non-survivors.

Parameter	Reference	Survivors	Non-Survivors	*p*-Value
Age (years)		73 (65; 78)	76 (67; 82)	0.354
Leukocytes (×10^9^/L)	6000–8000	14,970 (11,380; 18,620)	16,700 (13,700; 21,000)	0.148
FBG (mg/dL)	<125	126 (106; 165)	125 (110; 189)	0.429
AST (U/L)	10–35	23 (14; 36)	28 (19; 47)	0.364
ALT (U/L)	7–45	33 (17; 53)	40 (36; 78)	0.102
Urea (mg/dL)	17–49	61 (40; 86)	92 (67; 157)	0.011 *
Creatinine (mg/dL)	0.70–1.35	1.32 (0.84; 1.89)	1.96 (1.49; 2.64)	0.002 *
IL-6 (pg/mL)	<7	57 (23; 154)	190 (45; 340)	0.006 **
CRP (mg/L)	<5	177 (96; 242)	295 (175; 360)	0.021 *
ESR (mm/h)	<30	55 (40; 70)	66 (55; 92)	0.042 *
Procalcitonin (ng/mL)	0.05	1.68 (0.81; 4.59)	1.90 (0.91; 4.22)	0.921
CCI		5 (3; 6)	6 (4; 8)	0.010 *
Presepsin (pg/mL)	<300	1007 (670; 1564)	1446 (854; 2765)	0.039 *

FBG, fasting blood glucose; AST, aspartate aminotransferase; ALT, alanine aminotransferase; IL-6, interleukin-6; CRP, C-reactive protein; ESR, erythrocyte sedimentation rate; CCI, Charlson Comorbidity Index. Data are given as median with lower and upper quartiles in parentheses. Bold values marked with asterisks (*) indicate significant intergroup differences (Mann–Whitney tests with Benjamini–Hochberg correction, ***—*p* ≤ 0.001, **—*p* ≤ 0.01, *—*p* ≤ 0.05).

**Table 3:** Predictors of in-hospital mortality at admission in different regression models.

**Univariate Logistic Model**					
**Predictor**	**β**	**SE**	**OR (95% CI)**	**z**	***p***
AF	0.68	0.26	1.98 (1.19–3.29)	2.65	0.008 **
Urea	0.71	0.28	2.03 (1.16–3.56)	2.48	0.013 *
Creatinine	0.78	0.27	2.18 (1.26–3.76)	2.79	0.005 **
IL-6	1.04	0.65	2.82 (0.79–10.06)	1.62	0.108
CRP	0.71	0.29	2.03 (1.13–3.64)	2.39	0.017 *
ESR	0.28	0.14	1.32 (1.00–1.76)	1.99	0.047 *
CCI	0.64	0.28	1.91 (1.10–3.32)	2.30	0.021 *
Presepsin	0.51	0.24	1.66 (1.04–2.66)	2.10	0.035 *
**Multivariate Logistic Model**					
**Predictor**	**β**	**SE**	**AOR (95% CI)**	**z**	***p***
AF	0.57	0.25	1.76 (1.09–2.84)	2.31	0.021 *
Urea	0.01	0.01	1.01 (0.99–1.03)	0.41	0.659
Creatinine	0.34	0.17	1.40 (1.00–1.95)	1.98	0.048 *
CRP	0.02	0.01	1.02 (0.99–1.05)	1.17	0.256
ESR	0.01	0.01	1.00 (0.99–1.01)	0.66	0.503
CCI	0.11	0.16	1.12 (0.82–1.53)	0.72	0.466
Presepsin	0.01	0.01	1.00 (0.99–1.00)	0.76	0.447
**LASSO Regression Model**					
**Predictor**	**LASSO β**		**Selected (β ≠ 0)**	**BSF**	
AF	0.472		DA	**92.30% ^†^**	
Urea	0.209		DA	65.12%	
Creatinine	0.447		DA	**84.91% ^†^**	
CRP	0.320		DA	79.62%	
ESR	0.260		DA	78.93%	
CCI	0.298		DA	72.14%	
Presepsin	0.214		DA	74.52%	

IL-6, interleukin-6; CRP, C-reactive protein; ESR, erythrocyte sedimentation rate; CCI, Charlson Comorbidity Index; AF, atrial fibrillation; β, coefficient beta; SE, standard error; OR (95% CI), odds ratio with 95% confidence interval; AOR (95% CI), adjusted odds ratio with 95% confidence interval; Wald (Z), Z-value from the Wald test; *p*, *p* value; LASSO β, penalized regression coefficient derived from L1-regularized logistic regression; BSF, bootstrap selection frequency (1000 iterations); Selected (β ≠ 0), variables retained after penalization; BSF, bootstrap selection frequency based on 1000 resamples. Univariate odds ratios represent odds per one standard deviation increase; multivariate odds ratios represent odds per one-unit increase in original clinical measurement scales. Atrial fibrillation was modeled as a combined (pre-existing/new-onset) variable due to limited subtype-specific statistical power. Bold values marked with asterisks (*) in the last columns from the first two subtables indicate significant predictors of in-hospital mortality (Univariate/multivariate logistic regression, ***—*p* ≤ 0.001, **—*p* ≤ 0.01, and *—*p* ≤ 0.05). Bold values marked with dagger symbols (†) in the last column from the third subtable indicate robust predictors (BSF ≥ 80%) in the LASSO regression model.

**Table 4:** Comparative analysis of diagnostic performance and statistical validation of predictive models for mortality in sepsis.

Model	AUC (ROC)	Accuracy	Sensitivity	Specificity	NPV	Nag. R^2^	H-L (*p*)	DL (*p*)
AF	0.682 (0.580–0.784)	73.6%	60.0%	76.3%	90.6%	0.185	0.910	-
AF + CR	0.818 (0.731–0.905)	74.7%	80.0%	59.2%	94.9%	0.364	0.755	0.028 *
AF + CR + Urea	0.823 (0.739–0.907)	74.7%	86.7%	72.4%	96.5%	0.371	0.544	0.014 *
AF + CR + CRP	0.848 (0.772–0.924)	75.8%	86.7%	73.7%	96.6%	0.422	0.812	0.003 **
AF + CR + ESR	0.830 (0.748–0.912)	78.0%	80.0%	77.6%	95.2%	0.389	0.688	0.011 *
AF + CR + CCI	0.831 (0.750–0.912)	76.9%	80.0%	76.3%	95.1%	0.392	0.724	0.009 **
AF + CR + Presepsin	0.843 (0.764–0.922)	82.4%	80.0%	82.9%	95.5%	0.412	0.841	0.004 **

AUC (ROC), area under the receiver operating characteristic curve (ROC); Accuracy, overall classification accuracy; Sensitivity, true positive rate; Specificity, true negative rate; NPV, negative predictive value; Nag. R^2^, Nagelkerke pseudo-R^2^ indicating the proportion of variance explained by the model; H–L (*p*), *p*-value of the Hosmer–Lemeshow goodness-of-fit test; DL (p), *p*-value from the DeLong test comparing the AUC of each model with that of the reference model; AF, atrial fibrillation; CR, creatinine; CRP, C-reactive protein; ESR, erythrocyte sedimentation rate; CCI, Charlson Comorbidity Index. Atrial fibrillation was defined as a combined variable encompassing both pre-existing and new-onset AF identified at admission, irrespective of temporal onset. The AUC values in the second column are given as absolute values along with their corresponding 95% confidence intervals. Bold values marked with asterisks (*) indicate significant differences in AUC compared with AF (DeLong test, ***—*p* ≤ 0.001, **—*p* ≤ 0.01, *—*p* ≤ 0.05).

**Table 5:** Preliminary Sepsis CORE derivation score components and exploratory risk stratification framework.

**Defining Critical Thresholds**				
**Variable**	**Cut-Off**	**Score Points**	**Statistical Justification**	
AF	Present (prior history or new onset)	1	Cardiovascular susceptibility factor	
Creatinine	>1.49 mg/dL	1	Acute renal dysfunction marker	
Presepsin	>1446 pg/mL	1	Prognostic threshold of immune activation	
**Risk distribution by score levels**				
**Sepsis CORE Score**	**Number of** **patients (*n*)**	**Non-survivors (*n*)**	**Mortality** **(%)**	**Clinical interpretation**
0 Points	84	0	0%	Stable patient with favorable short-term prognosis. Standard monitoring recommended.
1 Points	48	4	8.3%	Closer monitoring and clinical reassessment may be appropriate due to potential deterioration risk
2 Points	30	14	46.7%	Substantial mortality risk. Closer monitoring and consideration of escalation of care may be warranted.
3 Points	20	12	60.0%	Extremely guarded prognosis. Urgent reassessment and consideration of higher-level monitoring or escalation of care may be warranted.

AF, atrial fibrillation. Data in the second and third columns are presented as absolute values, whereas data in the fourth column are presented as percentages.

## Data Availability

The original contributions presented in this study are included in the article. Further inquiries can be directed to the corresponding authors.

## Abbreviations

The following abbreviations are used in this manuscript:

ALT	Alanine aminotransferase
AOR	Adjusted odds ratio
AST	Aspartate aminotransferase
AUC	Area under the curve
CCI	Charlson Comorbidity Index
CI	Confidence interval
CKD	Chronic kidney disease
CRP	C-reactive protein
ED	Emergency Department
ESR	Erythrocyte sedimentation rate
FBG	Fasting blood glucose
AF	Atrial fibrillation
ICU	Intensive Care Unit
IL-6	Interleukin-6
IQR	Interquartile range
LASSO	Least Absolute Shrinkage and Selection Operator
MEWS	Modified Early Warning Score
NEWS2	National Early Warning Score 2
NPV	Negative Predictive Value
OR	Odds ratio
PCT	Procalcitonin
ROC	Receiver Operating Characteristic
SOFA	Sequential Organ Failure Assessment
